# Integrating Electrocardiography and Vectorcardiography in the Differential Diagnosis of Wide Complex Tachycardia in a Patient with Left Ventricular Noncompaction: A Case Report and Brief Literature Review

**DOI:** 10.3390/diagnostics11071152

**Published:** 2021-06-24

**Authors:** Călina-Patricia Țentea, Csilla-Andrea Eötvös, Roxana-Daiana Lazar, Iulia-Georgiana Zehan, Giorgia Paștiu, Adriana Porca, Mihaela Jelnean, Sorin Pop, Dan Blendea

**Affiliations:** 1 Cluj County Emergency Hospital, 400000 Cluj-Napoca, Romania; tenteapatricia@yahoo.it (C.-P.Ț.); daiana.pocol@yahoo.com (R.-D.L.); iuliazehan@gmail.com (I.-G.Z.); giorgia23pastiu@yahoo.com (G.P.); adriana_porca@yahoo.com (A.P.); mihaelajelnean@gmail.com (M.J.); popsorin98@gmail.com (S.P.); 2 Department of Medicine, Faculty of Medicine, University of Medicine and Pharmacy “Iuliu Hatieganu”, 400012 Cluj-Napoca, Romania; csilla.andrea18@gmail.com; 3 “Niculae Stancioiu” Heart Institute, 400001 Cluj-Napoca, Romania

**Keywords:** wide complex tachycardia, electrocardiogram, vectorcardiogam

## Abstract

A 69-year-old woman with a history of hypertension and obesity, hospitalized with atypical chest pain, was diagnosed with left ventricular noncompaction. In-hospital monitoring of the cardiac rhythm revealed multiple episodes of atrial tachycardia and one episode of wide complex tachycardia (WCT) with left bundle branch block-like morphology and a right superior QRS axis. The electrocardiographic criteria were suggestive of a supraventricular origin of the WCT. Given the importance of reaching the correct diagnosis when dealing with a WCT, we tried to further define the pattern of ventricular activation using vectorcardiography (VCG). We analyzed the QRS loops during WCT in comparison to a sinus beat, a narrow complex tachycardia beat, and a premature ventricular contraction. The fast initial activation seen in the efferent limb of the QRS loop during the WCT was thought to be reflective of the fast initial activation via the conduction system seen in SVT with aberrancy, which was our final diagnosis for the WCT episode. This case illustrates a novel use of vectorcardiography as an additional diagnostic tool in the differential diagnosis of WCT.

## 1. Introduction

Differential diagnosis of wide complex tachycardia (WCT) represents a challenge for many physicians, given the various electrocardiographic (ECG) criteria available and, importantly, the associated therapeutic consequences of inaccurate diagnosis. Left ventricular noncompaction (LVNC) is a cardiomyopathy associated with heart failure, thromboembolism, or arrhythmias, including sudden cardiac death [1]. Herein, we present a case of LVNC associated with atrial arrhythmias, as well as an episode of WCT with a challenging differential diagnosis. We used established ECG algorithms and criteria for the differential diagnosis of WCT, which was, in our case, supraventricular tachycardia (SVT) conducted with aberrancy. Given the clinical importance of excluding ventricular tachycardia (VT) with a reasonable degree of certainty, we describe a novel approach to delineate differences in ventricular activation velocity by vectorcardiography (VCG), and thus to confirm the diagnosis of SVT with aberrant conduction.

## 2. Case Presentation

A 69-year-old woman with a history of obesity and hypertension was hospitalized with atypical chest pain, palpitations, right upper quadrant pain, nausea, and weakness. Her medication included an ACE inhibitor, a thiazide-like diuretic, and aspirin. On arrival, her pulse was 94 beats/min, her blood pressure was 140/90 mmHg, and her oxygen saturation was 95% on room air. On physical examination, she appeared well, the skin was warm and dry, and there were no overt signs of congestive heart failure. 

### 2.1. Investigations

**Figure 1:**
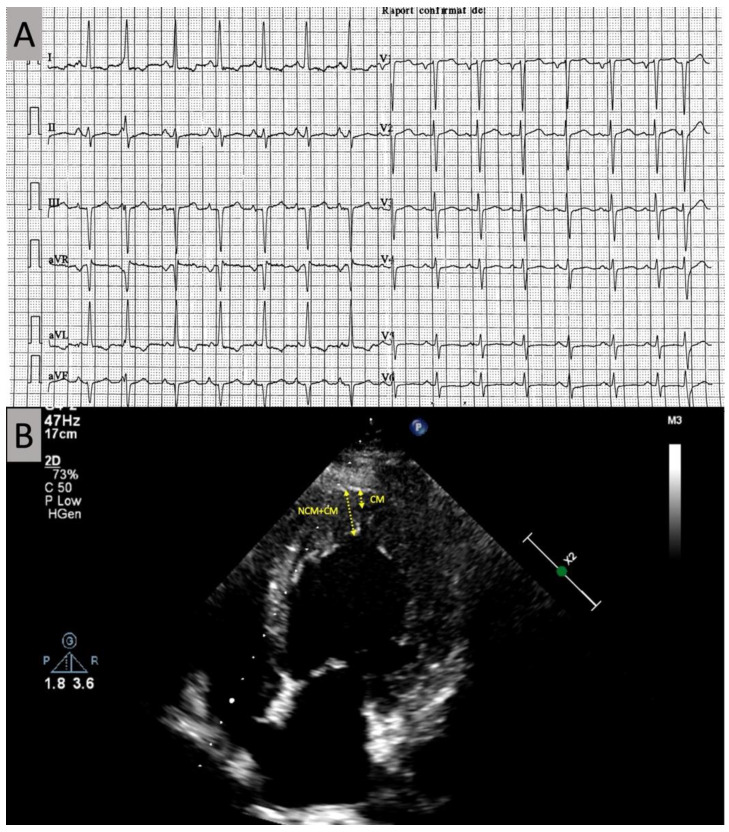
(**A**) Baseline electrocardiogram; (**B**) echocardiogram with increased trabeculation seen at the left ventricular apex. NCM, non-compact myocardial layer; CM, compact myocardial layer.

An electrocardiogram (Figure 1A) showed sinus rhythm, with premature junctional beats, left axis deviation (−31°), poor R wave progression in the precordial leads, bi-atrial enlargement, and left ventricular (LV) hypertrophy with secondary repolarization abnormality. 

The echocardiogram (Figure 1B) demonstrated diffuse hypokinesis, predominantly septal, with an LV ejection fraction of 37%, bi-atrial enlargement, mild-to-moderate mitral regurgitation, and LV hypertrophy, with a particular apical aspect of trabeculated myocardium, most prominent in the apex, apical segment of the septum, and distal lateral wall, raising the possibility of LVNC. A contrast echocardiography study revealed a noncompact/compact myocardium ratio greater than 2, measured at the end systole in the short-axis parasternal view, with deep endomyocardial recesses, diagnostic for LVNC according to the Jenni criteria [2].

Given the newly diagnosed cardiomyopathy with reduced systolic function, a coronary angiogram was performed and revealed coronary arteries with no significant stenoses. 

**Figure 2:**
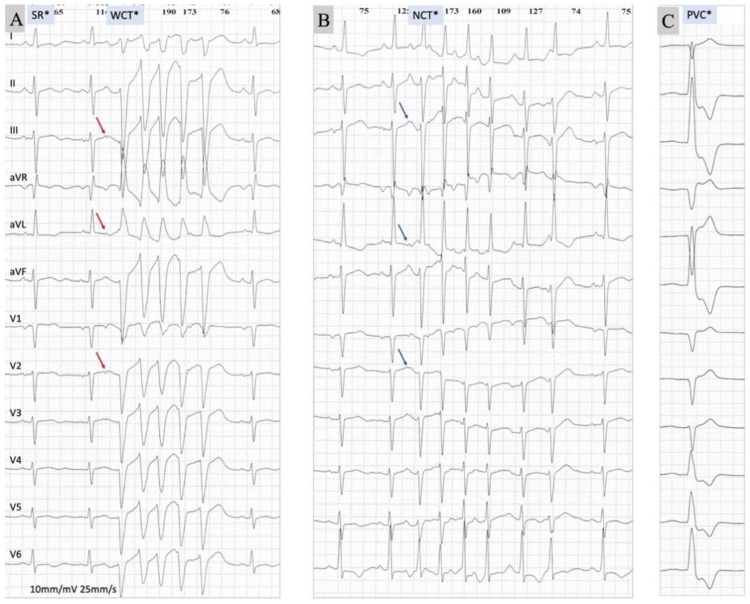
The 12-lead Holter recordings during (**A**) sinus rhythm (SR) and wide complex tachycardia (WCT), during (**B**) narrow complex tachycardia (NCT), and a during (**C**) premature ventricular contraction (PVC). Beats marked with an asterisk (SR*, WCT*, NCT*, or PVC*) are displayed as VCG loops in Figure 3.

The baseline rhythm was sinus, on a 48 h Holter recording that also demonstrated frequent premature atrial contractions, as well as multiple nonsustained runs of narrow complex tachycardia with an ECG pattern suggestive of SVT, likely atrial tachycardia. The recordings also included one episode of WCT with a QRS width of 150 ms, consisting of five beats, at a rate of 140 bpm, with left bundle branch block (LBBB)-like morphology and a right superior QRS axis (Figure 2). 

None of the Brugada or Vereckei criteria for VT were clearly present (Table 1). 

**Figure 3:**
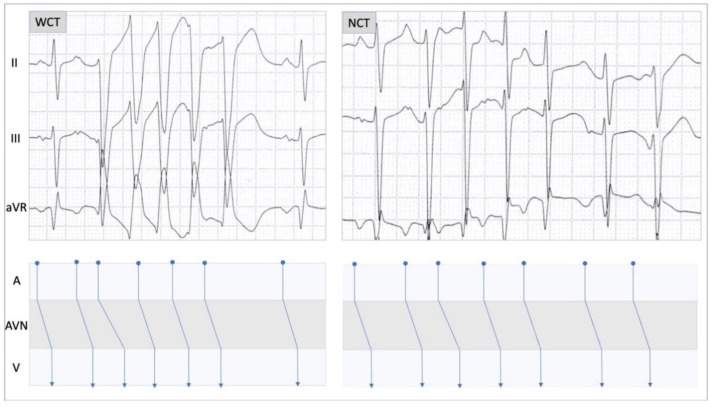
Ladder diagram illustrating the mechanism of conduction at the level of the atria (A), atrioventricular node (AVN), and ventricles (V). The left panel displays an episode of wide complex tachycardia (WCT) and the right panel shows an episode of narrow complex tachycardia (NCT). Note that both episodes start with a premature atrial contraction-conducted anterogradely via the AVN.

Analysis of the mode of initiation of the tachycardia episode for both WCT and NCT (Figure 3) showed a similar pattern present in both, with a premature atrial contraction initiating the WCT, as well as the narrow complex tachycardia (NCT). The ladder diagram revealed atrial beats preceding and driving ventricular beats during WCT and NCT, in a similar fashion. This ECG pattern is suggestive for atrial tachycardia with ventricular aberrancy in the case of WCT. 

**Figure 4:**
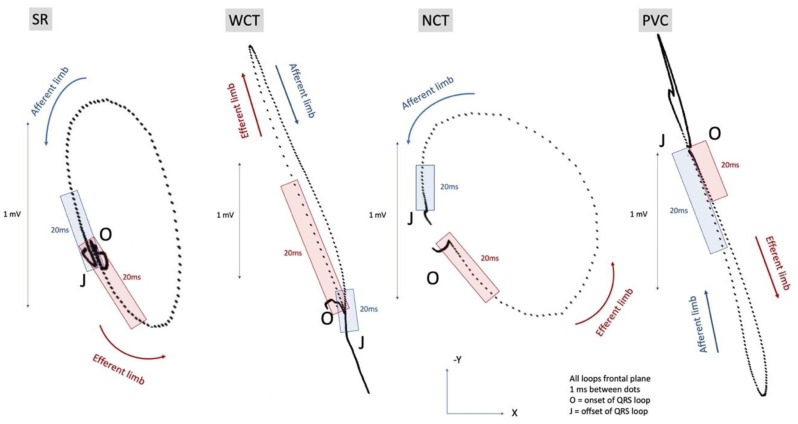
Vectocardiographic QRS loops in the frontal plane during sinus rhythm (SR), wide complex tachycardia (WCT), narrow complex tachycardia (NCT), and ventricular premature contraction (PVC). These are the beats displayed with an asterisk in Figure 2. The distance between black markers corresponds to 1 ms.

Given the importance of excluding VT, we tried to further define the pattern of ventricular activation during WCT using VCGs, which were generated from the 12-lead electrocardiographic monitoring tracings (Figure 4). We analyzed the QRS loops during WCT in comparison to a sinus beat, a narrow complex NCT beat, and a premature ventricular contraction (PVC).

The timing and sequence of ventricular activation at baseline (sinus beats with narrow QRS) showed similar velocities of activation during the efferent and the afferent limbs. During NCT, ventricular activation showed a similar pattern, albeit slightly faster during the initial 20 ms than during the terminal 20 ms. During WCT, ventricular activation in the initial portion of the efferent limb was significantly faster (more widely distanced 1 ms markers) than in the afferent limb. This contrasted with the slow initial activation (closely spaced 1 ms markers) seen during the PVC. 

The fast initial activation seen in the efferent limb of the QRS loop during the WCT was thought to be reflective of the fast initial activation via the conduction system seen in SVT with aberrancy, which was our final diagnosis for the WCT episode.

### 2.2. Management

The patient was treated for heart failure with reduced ejection fraction according to the current guidelines, with a combination of a betablocker, an ACE inhibitor, a mineralocorticoid antagonist, and a loop diuretic. On this regimen, the patient improved clinically and, at her 12-month follow-up visit, reported no interval hospitalizations. There were no further WCT episodes during Holter monitoring, and the echocardiogram revealed an improvement in the LV ejection fraction to 48%.

## 3. Discussion

Demonstrated here as a case of a patient newly diagnosed with LVNC, who developed wide complex tachycardia with LBBB-like morphology and a superior axis. The diagnosis wzs made using the Brugada and Vereckei algorithms, as well as by analyzing the mode of onset of tachycardia. We also described a novel approach to delineate differences in ventricular activation velocity by VCG analysis, which confirmed the diagnosis of SVT with aberrant conduction.

### 3.1. Atrial and Ventricular Arrhythmias in LVNC

The American Heart Association classifies LVNC as a distinct primary cardiomyopathy with a genetic etiology [3], characterized by a regional two-layered myocardium with a spongiform thickening of the endocardium and a compact thin epicardial layer [4,5]. LVNC is associated with heart failure, embolic events, and atrial and ventricular arrhythmias [1,6,7,8]. Ventricular activation may be impaired in LVNC due to the increased trabeculation associated with deep intramyocardial recesses. The Purkinje network in these patients is situated deeper into the mid-myocardium [9]. No specific ECG patterns have been described in patients with LVNC, although intraventricular conduction delay and aberrant ventricular conduction have been described [10,11].

The substrate for malignant ventricular arrhythmias and conduction disturbances in LVNC patients is multifactorial: Myocardial fibrosis, impaired flow reserve causing extended ischemia, and transmural heterogeneity with myocardial cells positioned around the deep intertrabecular recesses that may serve as slow conducting zones for re-entry [1]. Ventricular tachyarrhythmias increase the risk of sudden cardiac death in LVNC [12,13]. Ascertaining the presence or absence of VT in our patient was particularly important, especially since the patient had significant LV systolic dysfunction, which could also be a predictor for an increased risk of ventricular tachyarrhythmias [1,13,14,15].

### 3.2. Established Algorithms for the Differential Diagnosis of WCT

The WCT episode appeared more likely to be supraventricular in origin conducted with aberrancy. The two most established sets of criteria that help with the differential diagnosis of a WCT are included in the Brugada [16] and Vereckei [17] algorithms. None of the criteria for VT were clearly present. Nonetheless, VT could not be excluded with certainty because the presence of certain features used in the differential diagnosis of WCT [18,19] could not be clearly ascertained. Regarding the Brugada algorithm [16], there was no clear concordance, since an rS complex was present in the precordial leads, but the r waves were very small (0.1 mV or less in amplitude). The ventricular activation pattern in the precordial leads, with very small r waves and deep S waves throughout, resembled, to a certain extent, the ventricular concordance seen in VT. The other criteria for VT included in the Brugada algorithm were negative: The r to S interval was less than 100 ms and atrioventricular dissociation was not detected. 

None of the Verekei [17] criteria for VT were clearly present: There was no initial R wave in aVR, no initial q wave width of >40 ms, and no notching on the downstroke of a predominantly negative QRS complex. The ratio between the vertical excursion recorded during the initial (vi) and terminal (vt) 40 ms of the QRS complex (vi/vt) appeared to be >1; however, there was significant variability of this parameter from beat to beat. 

When we analyzed the onset of the WCT, we identified a premature atrial contraction (Figure 2, WCT*, red arrows) that appeared to be conducted with delay to the first wide QRS complex and to initiate the tachycardia episode [20]. In addition, this mode of tachycardia onset was similar to the onset of the episode of NCT (Figure 2, NCT*, blue arrows), which appeared to be triggered by a premature atrial contraction. This extrasystole was less premature than the one triggering the WCT episode, and the PR interval was longer than in sinus but shorter than the one at the onset of the WCT episode. Note that the sinus rate before the onset of WCT was slower than the rate that preceded the NCT episode, making a long–short sequence more likely in the case of the WCT. 

While most ECG features were suggestive of SVT, the QRS axis in the frontal plane during WCT was −100°, being in the “northwest” quadrant, which most of the time is associated with VT. To note, however, that the WCT was recorded using a Holter monitoring system, and the extreme left axis could, in part due to axis shifts caused by changes in body position during the monitoring period. The QRS axis of the sinus beat before the onset of WCT was −60°, which represents a 29° leftward shift from the ECG at baseline in the supine position (QRS axis −31°), very likely caused by a change in body position. 

Since the QRS axis and morphology seemed to be influenced, in part, by positional changes, we shifted our attention to the timing of ventricular activation in relationship to atrial activation to help clarify the WCT diagnosis.

### 3.3. Mode of Initiation of the WCT Episode

Perhaps one of the most compelling arguments for a supraventricular origin for the WCT episode in our patient was the analysis of the mode of tachycardia initiation (Figure 3). Comparative analysis of the onset of tachycardia revealed that both the WCT, as well as the NCT, were initiated in the same fashion by a premature atrial beat. In addition, the atrial beats, as seen on the ECG and depicted on the ladder diagrams, were driving the ventricular contractions, which is highly suggestive of supraventricular tachycardia. 

### 3.4. Vectorcardiographic Analysis of WCT: Differences in Ventricular Activation Velocity

Differences in ventricular activation velocity are extremely important when one is trying to discern the origin of a WCT. In SVT, initial activation is fast, via the conduction system, while in VT, it is slow because it occurs via muscle-to-muscle conduction. 

We used VCG, which is considered a complement to standard 12-lead ECG, offering a better insight into the spatial orientation and timing of the ventricular activation. VCG displays cardiac activation with a vector loop in three orthogonal planes—frontal (XY leads), horizontal (XZ leads), and sagittal (ZY leads)—and has been reported to be particularly helpful in defining ventricular activation in patients with conduction disease [21,22]. The progression of the instantaneous depolarization vector is represented on the QRS loop as markers (dots or arrows), equally distanced from a time perspective (1 ms between markers in our case). Closely spaced markers indicate a slow rate of inscription, while widely spaced markers indicate a rapid inscription of electrical activity. 

Several observational studies have tried to define the VCG patterns associated with PVCs or VT [22,23,24]. While the QRS loop morphology and vector orientation vary among different patients with VT, a VCG pattern uniformly described in patients with VT is the slow initial activation on the QRS loops (first 20 ms), as described by Talbot et al. [23,24]. Studies focused on VCG in aberrant ventricular conduction have revealed multiple patterns resembling the ones seen in bundle branch block and/or fascicular block [25]. The analysis of the vectorcardiograms during WCT in our patient did not reveal the slow initial activation on the QRS loops usually seen in PVCs and VT. This pattern was actually observed in our patient on the PVC vectorcardiogram (Figure 4, PVC panel). The ventricular activation during the first 20 ms of the QRS in our WCT case was fast (widely spaced markers in the efferent loop), even faster than during the 20 ms terminal. 

Note that the pattern seen in the sinus rhythm at baseline had markers relatively equally distanced on the afferent and efferent loops with fast ventricular activation via the conduction system during the initial as well as the 20 ms terminals of the QRS loop. 

During SVT with aberrancy, ventricular activation in the efferent loop appears to be faster than during sinus rhythm (in both cases via the conduction system), likely due to the fact that the amount of myocardium activated via the Purkinje network is significantly less than during sinus rhythm; therefore, it activates faster. 

Slow initial ventricular activation is one of the most important VCG criteria when diagnosing VT [23]. This is a reflection of the fact that during VT, the initial ventricular activation is as slow or slower than the terminal activation due to an initial slower muscle-to-muscle spread of activation. This is in contrast to the rapid initial activation in SVT with bundle branch block aberrancy, where widening of the QRS is caused by a delay in the mid-to-terminal part of the QRS, a phenomenon that has been incorporated into both the Vereckei and Brugada criteria for the differential diagnosis of the WCT [16,17].

Although comparative analysis of the speed of inscription in the initial portion of the QRS loop is based on a known pattern of ventricular activation in VT and SVT, the use of comparative analysis of the efferent and afferent limbs of the QRS loops, in addition to the more established ECG criteria for differentiating VT from SVT, is new. We believe that analysis of the VCG loop allows a better visualization of the three-dimensional geometry of the ventricular activation, which is ultimately one of the pillars in the SVT/VT differential.

Given that reconstructed VCGs are readily available in most ECG and Holter monitoring systems, the addition of VCG analysis in the differential diagnosis of WCT could prove to be helpful when analysis of a reconstructed VCG is available.

## 4. Conclusions

This case illustrated a novel use of vectorcardiography as an additional diagnostic tool in the differential diagnosis of WCT. If validated in future studies, this method could be incorporated into the diagnostic algorithms for WCT. This case also illustrated the need to integrate all information available and not to absolutize any criteria in the differential diagnosis process.

## Figures and Tables

**Table 1:** Applying the Brugada and Vereckei criteria for the episode of wide complex tachycardia detected during Holter monitoring.

Brugada Criteria for VT		Vereckei Criteria for VT	
Chest lead concordance	Not present (very small r waves)	AV dissociation present	Probably absent (P-QRS association at WCT onset and termination, but not all P waves seen)
RS interval >100 ms	Absent	Initial R wave in aVR	Absent
AV dissociation	Probably absent (P-QRS association at WCT onset and termination, but not all P waves seen)	QRS morphology unlike bundle branch block or fascicular block	Absent
VT morphology criteria in V1–V2, V6	Absent	V_i_/V_t_ ≤ 1	Absent

VT, ventricular tachycardia; AV, atrioventricular. The ratio between the vertical excursion recorded during the initial (V_i_) and terminal (V_t_) 40 ms of the QRS complex (V_i_/V_t_) appeared to be >1 in our patient.
